# SPR Biosensor Probing the Interactions between TIMP-3 and Heparin/GAGs

**DOI:** 10.3390/bios5030500

**Published:** 2015-07-23

**Authors:** Fuming Zhang, Kyung Bok Lee, Robert J. Linhardt

**Affiliations:** 1 Center for Biotechnology and Interdisciplinary Studies, Department of Chemical and Biological Engineering, Rensselaer Polytechnic Institute, Troy, NY 12180, USA; 2 Department of Biochemistry, College of Medicine, Konyang University, Konyang Hospital, Daejeon 302-718, Korea; E-Mail: kyunglee@konyang.ac.kr; 3 Center for Biotechnology and Interdisciplinary Studies, Department of Chemistry and Chemical Biology, Rensselaer Polytechnic Institute, Troy, NY 12180, USA; 4 Departments of Biomedical Engineering, and Biological Sciences, Center for Biotechnology and Interdisciplinary Studies, Rensselaer Polytechnic Institute, Troy, NY 12180, USA

**Keywords:** heparin, glycosaminoglycans, TIMP-3, binding, surface plasmon resonance

## Abstract

Tissue inhibitor of metalloproteinases-3 (TIMP-3) belongs to a family of proteins that regulate the activity of matrix metalloproteinases (MMPs), which can process various bioactive molecules such as cell surface receptors, chemokines, and cytokines. Glycosaminoglycans (GAGs) interact with a number of proteins, thereby playing an essential role in the regulation of many physiological/patho-physiological processes. Both GAGs and TIMP/MMPs play a major role in many cell biological processes, including cell proliferation, migration, differentiation, angiogenesis, apoptosis, and host defense. In this report, a heparin biosensor was used to map the interaction between TIMP-3 and heparin and other GAGs by surface plasmon resonance spectroscopy. These studies show that TIMP-3 is a heparin-binding protein with an affinity of ~59 nM. Competition surface plasmon resonance analysis indicates that the interaction between TIMP-3 and heparin is chain-length dependent, and *N*-sulfo and 6-*O*-sulfo groups (rather than the 2-*O*-sulfo groups) in heparin are important in the interaction of heparin with TIMP-3. Other GAGs (including chondroitin sulfate (CS) type E (CS-E)and CS type B (CS-B)demonstrated strong binding to TIMP-3, while heparan sulfate (HS), CS type A (CSA), CS type C (CSC), and CS type D (CSD) displayed only weak binding affinity.

## 1. Introduction

The surface plasmon resonance (SPR) spectrometer is based on an optical biosensor technique that measures molecular binding events on a metal surface by detecting changes in the local refractive index [1]. Although SPR is a relatively new biophysical method, with the first commercial instrument being released from Biacore in 1990 [2], it has become a powerful tool to measure biomolecular interactions in real-time and in a label-free environment. SPR has been applied to characterize the binding events with samples ranging from proteins, nucleic acids, carbohydrates (glycans), and small molecules to complex mixtures, lipid vesicles, viruses, bacteria, and eukaryotic cells [3]. The use of SPR is increasingly popular in fundamental biological studies, health science research, drug discovery, clinical diagnosis, and environmental and bio-pharmaceutical process monitoring [4,5,6,7,8,9,10,11,12,13].

The tissue inhibitors of metalloproteinases (TIMPs) are a family of four homologous proteins that regulate the activity of matrix metalloproteinases (MMPs), and which are capable of degrading all kinds of extracellular matrix proteins and play important roles in many cell biological processes, such as cell proliferation, migration, differentiation, angiogenesis, apoptosis, and host defense [14]. In addition, TIMPs also possess growth stimulatory and regulatory activities [15,16]. Glycosaminoglycans (GAG) are anionic, polydisperse, linear polysaccharides that are often highly sulfated. GAGs include heparan sulfate (HS)/heparin, chondroitin sulfate/dermatan sulfate (CS/DS), hyaluronic acid (HA), and keratan sulfate. Interactions between heparin, heparan sulfate (HS), and other GAGs with proteins mediate diverse biological processes including: blood coagulation, cell growth and differentiation, host defense and viral infection, lipid transport and metabolism, cell-to-cell and cell-to-matrix signaling, inflammation, and cancer [17,18,19]. In our previous studies, we found that the sulfated GAGs play important roles in controlling both the activation and activity of MMP-7 [20,21]. There are reports showing that TIMP-3 interacts with GAGs such as heparin, heparan sulfate, chondroitin sulfates A, B, and C, and other sulfated compounds such as suramin and pentosan [14,22,23]. While these studies have provided general information on TIMP-3-GAG interactions, there is a lack of specific structural and biophysical data of these interactions.

The goal of this study is to analyze molecular interactions of TIMP-3 and heparin/GAGs. The BIAcore system (BIAcore 3000) was employed for the study. The system utilizes a heparin SPR biochip, which allows a direct quantitative analysis on molecular-level kinetic and structural requirements of the TIMP-3-GAG interaction.

## 2. Materials and Methods

### 2.1. Materials

**Figure 1:**
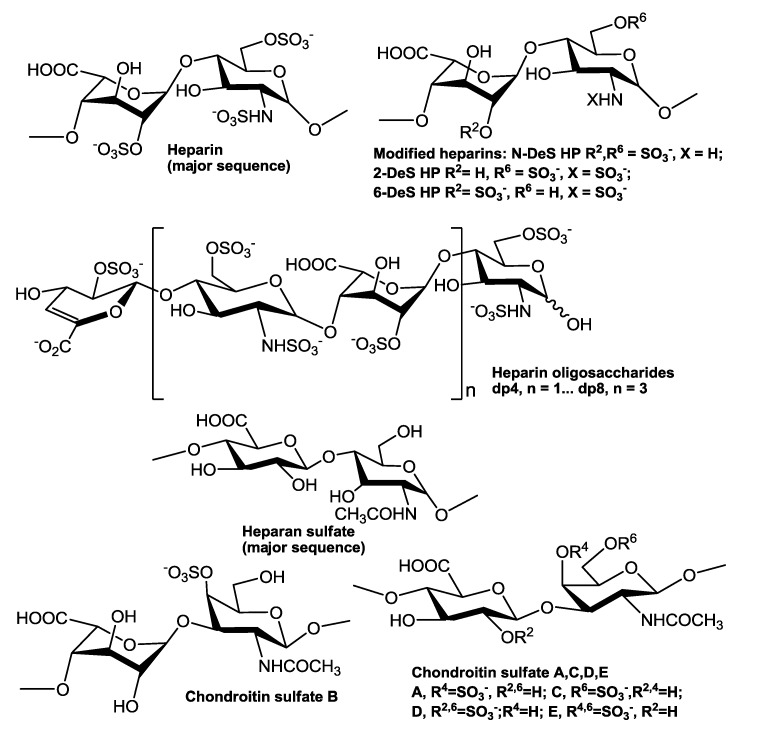
Chemical structures of heparin, heparin-derived oligosaccharides, chemically modified heparins, and other GAGs.

Human tissue inhibitor of metalloproteinases-3 (TIMP-3) was purchased from R&D systems (Minneapolis, MN, USA). The GAGs used include porcine intestinal heparin (16 kDa) and porcine intestinal heparan sulfate (Celsus Laboratories, Cincinnati, OH); chondroitin sulfate type A (20 kDa) from porcine rib cartilage (Sigma, St. Louis, MO), chondroitin sulfate type B, 30 kDa, from porcine intestine (Sigma); chondroitin sulfate type C (20 kDa, from shark cartilage, Sigma); chondroitin sulfate type D (20 kDa, from whale cartilage, Seikagaku, Tokyo, Japan); and chondroitin sulfate type E (20 kDa from squid cartilage, Seikagaku). *N*-desulfated heparin (14 kDa) and 2-*O*-desulfated IdoA heparin (13 kDa) were all prepared based on Yates *et al.* [24]. 6-*O*-desulfated heparin (13 kDa) was a generous gift from Dr. Lianchun Wang of the Complex Carbohydrate Research Center, University of Georgia [25]. Heparin oligosaccharides including disaccharide (dp2), tetrasaccharide (dp4), hexasaccharide (dp6), and octasaccharide (dp8) were prepared from controlled partial heparin lyase 1 treatment of bovine lung heparin (Sigma) followed by size fractionation. Chemical structures of these GAGs and heparin oligosaccharides are showed in Figure 1. Sensor streptavidin (SA) chips were from GE healthcare (Biacore AB, Uppsala, Sweden). SPR measurements were performed on a BIAcore 3000 operated using BIAcore 3000 control and BIAevaluation software (version 4.0.1) from GE healthcare.

### 2.2. Preparation of Heparin Biochip

The biotinylated heparin was prepared by the reaction of sulfo-*N*-hydroxysuccinimide long-chain biotin (Piece, Rockford, IL, USA) with free amino groups of unsubstituted glucosamine residues in the polysaccharide chain following a published procedure [26]. The biotinylated heparin was immobilized to the streptavidin (SA) chip based on the manufacturer’s protocol. In brief, 20 μl solution of the heparin-biotin conjugate (0.1 mg/mL) in HBS-EP running buffer was injected over flow cell 2 (FC2) of the SA chip at a flow rate of 10 μL/min. The successful immobilization of heparin was confirmed by the observation of a ~50 resonance unit (RU) increase in the sensor chip. The control flow cell (FC1) was prepared by 1 min injection with saturated biotin.

### 2.3. Measurement of Interaction between Heparin and TIMP-3 Using BIAcore

The protein samples were diluted in HBS-EP buffer (0.01 M HEPES, 0.15 M NaCl, 3 mM EDTA, 0.005% surfactant P20, pH 7.4). Different dilutions of protein samples were injected at a flow rate of 30 µL/min. At the end of the sample injection, the same buffer was flowed over the sensor surface to facilitate dissociation. After a 3 min dissociation time, the sensor surface was completely regenerated by injecting with 30 µL of 2 M NaCl. The response was monitored as a function of time (sensorgram) at 25 °C.

### 2.4. Solution Competition Study between Heparin on Chip Surface and Heparin-Derived Oligosaccharides in Solution Using SPR

**Figure 2:**
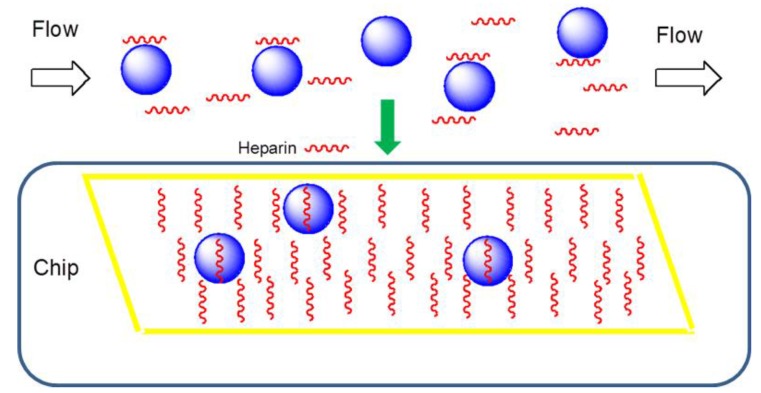
Diagram of SPR solution competition study. The blue spheres represent TIMP-3 protein, the short red helices represent heparin or other GAGs. TIMP-3 protein (pre-mixed with 1000 nM heparin oligosaccharides or GAG) was injected over a chip containing immobilized heparin; only free protein can bind to the heparin on the chip.

Solution/surface competition experiments were performed by SPR to examine the effect of the saccharide chain size of heparin on the heparin-protein interaction (Figure 2). In brief, TIMP-3 protein (10 nM) mixed with 1000 nM of heparin oligosaccharide, including disaccharide (dp2), tetrasaccharide (dp4), hexasaccharide (dp6), and octasaccharide (dp8), in HBS-EP buffer were injected over the heparin chip at a flow rate of 30 μL/min, respectively. After each run, the dissociation and the regeneration were performed as described above. For each set of competition experiments on SPR, a control experiment (only protein without any heparin or oligosaccharides) was performed to make sure the surface was completely regenerated and that the results obtained between runs were comparable.

### 2.5. Solution Competition Study between Heparin on Chip Surface and GAGs, Chemically Modified Heparin in Solution Using SPR

For testing the inhibition of other GAGs and chemically modified heparins to the TIMP-3-heparin interaction, TIMP-3 at 10 nM was pre-mixed with 100 nM of GAG or chemically modified heparin and injected over the heparin chip at a flow-rate of 30 μL/min. After each run, a dissociation period and regeneration protocol was performed as described above.

## 3. Results and Discussion

### 3.1. Kinetics Measurement of TIMP-3-Heparin Interactions

**Figure 3:**
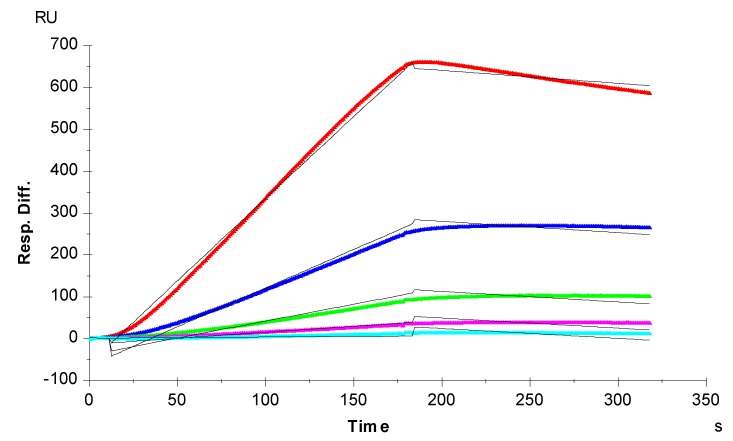
SPR sensorgrams of the TIMP-3-heparin interaction. Concentrations of TIMP-3 (from top to bottom): 20, 10, 5, 2.5, and 1.25 nM, respectively. The black curves are the fitting curves using the 1:1 Langmuir binding model from BIAevaluate 4.0.1.

Sensorgrams of TIMP-3-heparin interactions were obtained by injecting different concentrations of TIMP-3 on a heparin biosensor to afford binding kinetics (Figure 3). The sensorgrams at various TIMP-3 concentrations were globally fit by a 1:1 Langmuir model to obtain the binding kinetics (Table 1). The interaction of TIMP-3 to heparin displays a fast on-rate (*k_a_*) of 1800 M^−1^s^−1^ and slow off-rate (*k_d_*) of 1.1 × 10^−4^ s^−1^. The binding equilibrium dissociation constant (K_D_ = *k_d_/k_a_*) for the TIMP-3-heparin interaction is ~59 nM, confirming that TIMP-3 is a heparin-binding protein. This measured TIMP-3 binding affinity (K_D_) is comparable to the K_D_ recently reported by Troeberg *et al.* [22]. They also used SPR to measure the heparin-TIMP-3 binding affinity, but they immobilized TIMP-3 on the chip and flowed heparin/GAGs over the chip surface. It is worth nothing that in natural biological systems, most of GAGs are found attached on the cell surface through core proteins, and capture the target proteins that flow over the cell surface. Therefore, the measurement of GAG-protein interactions using SPR should closely mimic the real biological system where heparin is immobilized on the surface of a biosensor chip.

**Table 1:** Summary of kinetic data of TIMP-3/heparin interactions ^*^.

Interaction	k_a_ *(1/MS)*	k_d_ *(1/S)*	*K_D_ (M)*
TIMP-3/Heparin	1800 (±93)	1.1 × 10^−4^ (±1.4 × 10^−5^)	5.9 × 10^−8^

*The data with (±) in parentheses are the standard deviations (SD) from global fitting of five injections.

### 3.2. Solution Competition SPR Study Using Heparin-Derived Oligosaccharides

**Figure 4:**
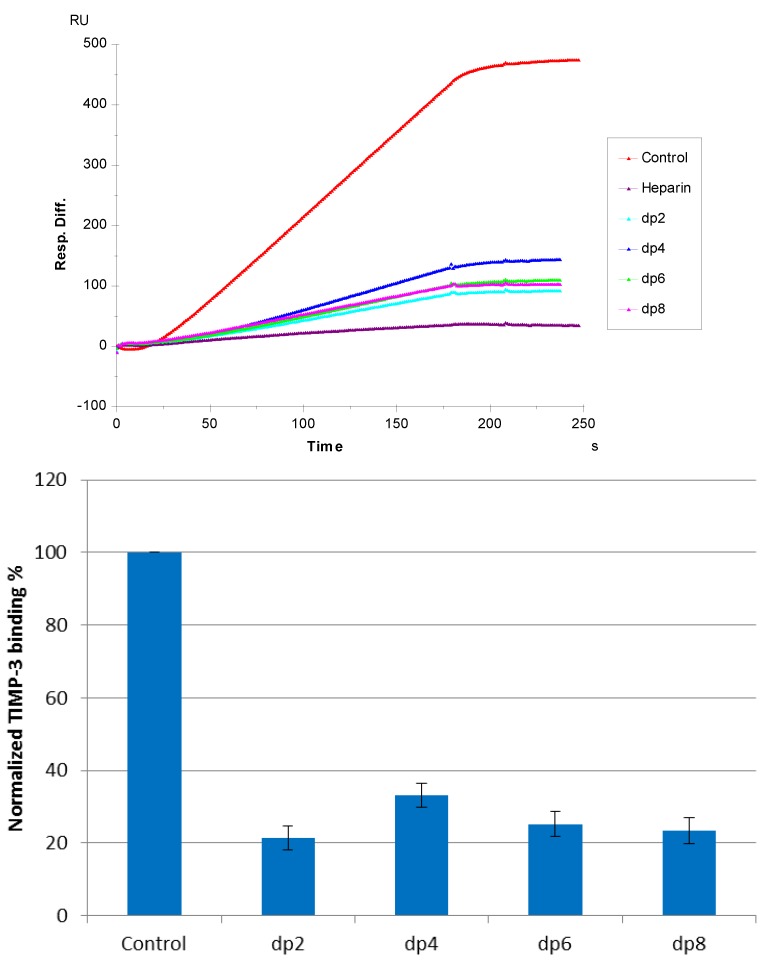
(**Top**): Sensorgrams of solution heparin oligosaccharides/surface heparin competition. TIMP-3 concentration was 10 nM, and concentrations of heparin oligosaccharides in solution were 1000 nM. (**Bottom**): Bar graphs (based on triplicate experiments with standard deviation) of normalized TIMP-3 binding preference to surface heparin by competing with different sizes of heparin oligosaccharides in solution.

Solution/surface competition experiments were performed by SPR to examine the effect of the saccharide chain size of heparin on the heparin-protein interaction. Different sizes of heparin-derived oligosaccharides (from dp2 to dp8) were used in the competition study. The same concentration (1000 nM) of heparin oligosaccharides were present in the TIMP-3 (10 nM)/heparin interaction solution. Obvious competition effect was observed (Figure 4) when 1000 nM of oligosaccharides (from dp 2 to dp8), present in the protein solution, suggests that the interaction between TIMP-3 and heparin does not require a long chain size of heparin. For heparin-protein interaction, there is usually a minimum chain size/length of heparin required. We previously reported that in sonic hedgehog heparin and interleukin 7-heparin interactions, the minimum chain size/length of heparin was octasaccharide and tetrasaccharide, respectively [27,28]. The chain size requirement for the interaction between TIMP-3 and heparin is shorter than those observed previously. It appears that an oligosaccharide as small as a disaccharide (dp2) may suffice in neutralizing the positive-charged heparin binding regions of TIMP-3 and compete successfully with immobilized heparin for TIMP-3 binding.

### 3.3. SPR Solution Competition Study of Different GAGs

**Figure 5:**
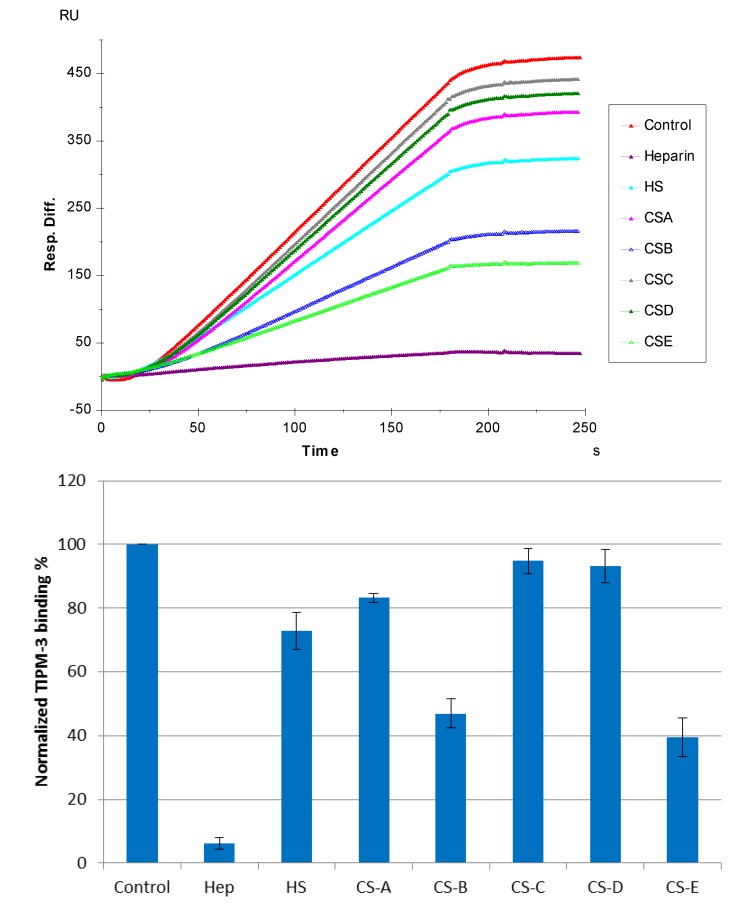
(**Top**): Sensorgrams of solution GAGs/surface heparin competition. TIMP-3 concentration was 10 nM, and concentrations of GAGs in solution were 100 nM. (**Bottom**): Bar graphs (based on triplicate experiments with standard deviation) of normalized TIMP-3 binding preferences to surface heparin by competing with different GAGs.

The SPR competition assay was also utilized to determine the binding preference of TIMP-3 to various GAGs (Figure 1). SPR competition sensorgrams and bar graphs of the GAG competition levels are displayed in Figure 5. CS-E and CS-B produced a strong inhibition by competing >60% and >50% of the TIMP-3 binding to immobilized heparin on the chip surface. Weak inhibitory activities were observed for HS, CS-A, CS-C, and CS-D. These results agree with Yu *et al.*, their report showing that TIMP-3 binds to heparin, heparan sulfate, and CS-A, B, and C [14].

### 3.4. SPR Solution Competition Study of Different Chemically Modified Heparins

**Figure 6:**
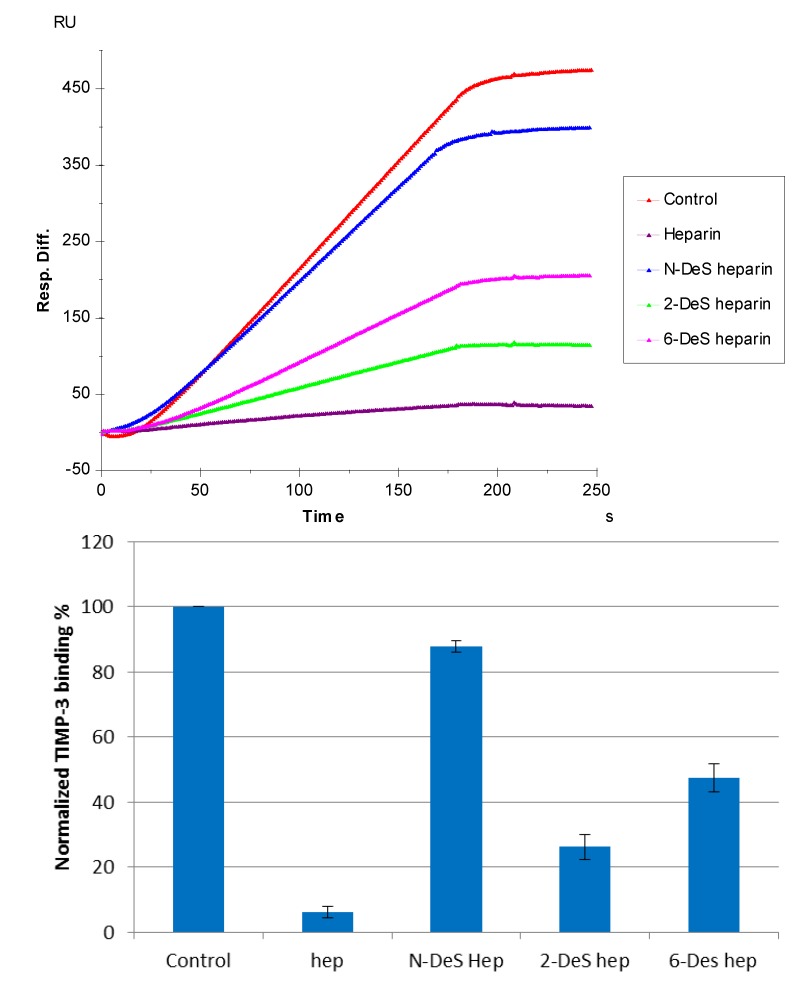
(**Top**): Sensorgrams of solution competition SPR using chemically modified heparin. TIMP-3 concentration was 10 nM, and concentrations of chemically modified heparin in solution were 1000 nM. (**Bottom**): Bar graphs (based on triplicate experiments with standard deviation) of normalized TIMP-3 binding preferences to surface heparin by competing with different chemically modified heparin in solution.

SPR competition sensorgrams and bar graphs of the chemically modified heparin competition levels are displayed in Figure 6. The results show that all three chemically modified heparins (*N*-desulfated heparin, 2-*O*-desulfated heparin, and 6-*O*-desulfated heparin) showed reduced inhibitory activities. Much higher reduced inhibitory activities were observed for 2-*O*-desulfated heparin and 6-*O*-desulfated heparin than for *N*-desulfated heparin, suggesting 2-*O*-sulfo and 6-*O*-sulfo groups on heparin have less impact on the TIMP-3-heparin interaction.

SPR competition experiments with different GAGs and chemically modified heparin showed the binding of TIMP-3 to GAGs was dependent on the level of GAG sulfation and fine structure. The competition effects of soluble GAGs to the immobilized heparin (chip surface) was greatest for heparin with 2.8 moles of sulfate per disaccharide repeating unit, followed by CS-E with 2 moles of sulfate per disaccharide. These results are similar to the GAG sulfation preference of most other proteins, including sonic hedgehog, interleukin 7, acidic fibroblast growth factor, and basic fibroblast growth factor, by interacting with more highly sulfated heparin than the less-sulfated HS [17,27,28]. Most surprising is the observation that a heparin disaccharide was capable of successfully competing with immobilized heparin for TEMP-3 binding.

The structural biology of MMP-TIMP interactions leading to MMP inhibition has been intensively studied [29,30]. MMPs are also involved with functional interactions with GAGs within the extracellular matrix leading to their activation [20,21,31]. TIMP-3, an inhibitor of all MMPs, also inhibits other metalloproteinases, such as disintigrin and metalloprotease (ADAM) and ADAM with thrombospondin motifs (ADMTS) [32]. ADAM17 catalyzes the release of tumor necrosis factor-α (TNF-α) [33]. The binding of TIMP-3 to a small, highly charged molecule, such as a heparin disaccharide, might alter its ability to inhibit ADAM17, offering a means to control the release of TNF-α. TIMP-3 only weakly binds to and inhibits ADAMTS, but this binding can be greatly enhanced through the chondroitin sulfate chains of aggrecan [34], heparin [35], and the synthetic GAG analog pentosanpolysulfate [36]. Since a heparin disaccharide is capable of blocking the interaction of heparin with TEMP-3, it is possible that small, highly charged analogs might be designed as novel therapeutic agents to regulate the activity of these metalloproteases.

## Conclusions

In conclusion, the heparin biosensor was successfully used to map the interaction between TIMP-3 and heparin and other GAGs by surface plasmon resonance spectroscopy, providing the kinetic and structural details of TIMP-3-GAG interactions. The understanding of interactions between these important biomolecules on a molecular level is of fundamental importance to the development of highly specific therapeutic agents.

## Abbreviations

TIMP-3: tissue inhibitor of metalloproteinases-3
GAG: glycosaminoglycan
MMP: matrix metalloproteinase
SPR: surface plasmon resonance
HS: heparan sulfate
CS-A: chondroitin sulfate type A
CS-B: chondroitin sulfate type B
CS-C: chondroitin sulfate type C
CS-D: chondroitin sulfate type D
CS-E: chondroitin sulfate type E
SA: streptavidin
FC: flow-cell
RU: resonance unit
dp: degree of polymerization
ADAM: disintigrin and metalloprotease
ADMTS: ADAM with thrombospondin motifs
TNF-α: tumor necrosis factor-α
